# The use of pituitary adenylate cyclase-activating polypeptide in the pre-maturation system improves *in vitro* developmental competence from small follicles of porcine oocytes

**DOI:** 10.5713/ajas.19.0162

**Published:** 2019-08-03

**Authors:** Kyu-Mi Park, Kyu-Jun Kim, Minghui Jin, Yongquan Han, Kyoung-Ha So, Sang-Hwan Hyun

**Affiliations:** 1 Institute for Stem Cell and Regenerative Medicine (ISCRM), Chungbuk National University, Cheongju 28644, Korea; 2 Laboratory of Veterinary Embryology and Biotechnology (VETEMBIO), Veterinary Medical Center and College of Veterinary Medicine, Chungbuk National University, Cheongju 28644, Korea

**Keywords:** Oocyte Maturation, Porcine, Follicles, Embryo Development, Apoptosis

## Abstract

**Objective:**

We investigated how pituitary adenylate cyclase-activating polypeptide (PACAP) affects embryonic development during pre-*in vitro* maturation (pre-IVM) using porcine oocytes isolated from small follicles.

**Methods:**

We divided the follicles into the experimental groups by size (SF, small follicles; MF, medium follicles) and treated with and without PACAP and cultured for 18 hours (Pre-SF[−]PACAP; without PACAP, Pre-SF[+]PACAP; with PACAP) before undergoing IVM. The gene expression related to extracellular matrix formation (amphiregulin, epiregulin, and hyaluronan synthase 2 [*HAS2*]) and apoptosis (Bcl-2-associated X [*BAX*], B-cell lymphoma 2, and cysteine-aspartic acid protease 3) was investigated after maturation. The impact on developmental competence was assessed by the cleavage and blastocyst rate and total cell number of blastocysts in embryos generated from parthenogenesis (PA) and *in vitro* fertilization (IVF).

**Results:**

Cleavage rates in the Pre-SF(+)PACAP after PA were significantly higher than SF and Pre-SF(−)PACAP (p<0.05). The cleavage rates between MF and Pre- SF(+)PACAP groups yielded no notable differences after IVF. Pre-SF(+)PACAP displayed the higher rate of blastocyst formation and greater total cell number than SF and Pre-SF(−)PACAP (p<0.05). Cumulus cells showed significant upregulation of *HAS2* mRNA in the Pre-SF(+)PACAP compared to the SF (p<0.05). In comparison to other groups, the Pre-SF(+)PACAP group displayed a downregulation in mRNA expression of *BAX* in matured oocytes (p<0.05).

**Conclusion:**

The PACAP treatment during pre-IVM improved the developmental potential of porcine oocytes derived from SF by regulating cumulus expansion and apoptosis of oocytes.

## INTRODUCTION

Despite a global increase in the use of assisted reproductive technologies (ART), obtaining oocytes of sufficient quality and number remains a limiting factor to ART efficiency in transgenic livestock production and human infertility treatments [1]. Among available ART, *in vitro* maturation (IVM) is most common, but oocytes developed through this method have elevated developmental incompetence, likely due to insufficient cytoplasmic maturation and nuclear abnormality [2], apparently associated with the dynamics of altered microtubules and chromatin phosphorylation [3].

Cumulus cells are also affected by *in vitro* environmental conditions. These cells are crucial in providing regulatory signals that control oocyte maturation and energy levels [4,5]. Their expansion is triggered by growth factor stimulation and accompanies extracellular matrix modification. These steps are necessary for ovulation and fertilization, including sperm acrosomal reactions. Cumulus expansion also influences the degree of protection against environmental stress, a factor linked to apoptosis. *In-vitro* and *in-vivo* matured oocytes have significantly more cumulus-cell-produced transcripts than *in-vitro* matured oocytes, indicating a clear difference in developmental competence and quality [6]. New IVM systems and improved IVM media have been developed to overcome these limitations [7]. Importantly, efforts should focus on minimizing atresia or reproductive defects to increase oocyte developmental capability [8].

Simulated physiological oocyte maturation (SPOM) is a promising new IVM system that uses cyclic adenosine 3′5′-monophosphate (cAMP) to improve oocyte capacitation [8]. The regulatory role of cAMP in nuclear maturation is well established [9–11]. Follicle-generated cAMP moves through gap junctions between cumulus cells and oocytes to reach the latter. In oocytes, cAMP stimulates protein kinase A (PKA) to prevent germinal vesicle breakdown; therefore, downregulating cAMP expression increases that breakdown. Inactivating PKA triggers meiosis resumption in oocytes and activates maturation promoting factor (MPF).

Under *in vivo* conditions, oocytes with meiotic competence have all the necessary proteins for meiosis and survival during embryonic development by the end of folliculogenesis [12]. However, when oocytes are artificially removed from follicles, cAMP levels decrease because the supply from follicles is no longer available. As a result, spontaneous nuclear maturation occurs. Two systems of SPOM have been developed to prevent this problem [13]. The first supplies a cAMP modulator to delay meiotic kinetics during pre-IVM. Subsequently during IVM, oocytes are treated with a phosphodiesterase 3 inhibitor. The second system involves supplementing oocytes with cAMP modulators during the pre-IVM phase only. Many studies have shown a positive effect with the letter system [9, 14,15]. Because this supplementation inhibits spontaneous meiotic processes of oocytes during pre-IVM, nuclear and cytoplasmic maturation then become synchronized during IVM, improving efficiency [10,14,16]. In cattle and mice, pre-IVM cAMP-modulator treatment improved embryo yield and subsequent development, including fetal yield, weight, and implantation [8,15].

Because they are based on oocytes derived from medium follicles (MF; 3 to 6 mm in diameter), standard IVM protocols fail to yield sufficient mature oocytes from small follicles (SF; ≤3 mm in diameter) [17]. Even those that reach metaphase II experience insufficient cytoplasmic maturation that causes low developmental competence [12,17]. This inefficiency is a problem because most of the follicles in large animals are small (early antral follicles) and there therefore discarded. As this is a waste of valuable genetic resources, there is considerable interest in using SPOM to improve SF-derived oocyte competence [18].

Hypothalamus-derived pituitary adenylate cyclase-activating polypeptide (PACAP) is a common cAMP activator that stimulates cells to produce cAMP. As a member of the secretin/glucagon/vasoactive intestinal peptide (VIP)/growth hormone releasing hormone family, PACAP exists in two forms, one with 38 amino acids (PACAP 38) and the other with 27 (PACAP 27) [19]. Of its three receptors (vasoactive intestinal polypeptide receptor 1 [VIPR1], VIPR2, and PAC1), PAC1 has the highest binding affinity, 1,000-fold greater than either VIP, with similar affinity to each other. A wide range of organs express PACAP and its receptors due to their roles in regulating apoptosis, inflammation, cell proliferation, and cell differentiation [20]. Recent studies have demonstrated the effects of PACAP on fertility and reproduction [21], possibly due to variation in PACAP action across cell types. Unlike the cAMP modulator commonly used in SPOM, PACAP may affect oocyte maturation during pre-IVM through binding to its receptor. In our previous research, we confirmed PACAP preliminary effects on oocyte maturation when administered pre-IVM [16]. We demonstrated that PACAP and its receptors were expressed in cumulus cells and oocytes. Furthermore, pre-IVM treatment with PACAP improved meiotic and cytoplasmic maturation through regulating intracellular oxidative stress. Our results led us to propose an optimal pre-IVM treatment duration and PACAP concentration for SF-derived oocytes.

In this study, our objective was to build on our previous work and determine whether PACAP-induced improvements to oocyte maturation are correlated with their developmental potential in IVF and parthenogenetic activation (PA). Using SF-derived porcine oocytes, we observed post-IVM expression of genes related to extracellular matrix formation (amphiregulin [*AREG*], epiregulin [*EREG*], and hyaluronan synthase 2 [*HAS2*]), apoptosis (Bcl-2-associated X [*BAX*], B-cell lymphoma 2 [*BCL-2*], and cysteine-aspartic acid protease 3 [*CASPASE-3*]), cell metabolism (hexokinase 2 [*HK2*]), and developmental competence (POU class 5 homeobox 1 [POU5F1]). Developmental potential was assessed via oocyte cleavage rate, blastocyst rate, and total blastocyst cell numbers.

## MATERIALS AND METHODS

### Chemicals

All chemicals and reagents in this study were purchased from Sigma-Aldrich Chemical Company (St. Louis, MO, USA), unless otherwise noted.

### Ovary collection and classification

Porcine ovaries were acquired from a local slaughter house and carried to the laboratory in 0.9% (w/v) NaCl solution between 32°C to 35°C. Cumulus-oocyte complexes (COCs) were separated based on their folliclular diameters: small (1 to 3 mm, SF) and medium (3 to 6 mm, MF). The COCs were aspirated using aspiration method. After washing with 4-(2-hydroxyethyl)-1-piperazineethanesulfonic acid-buffered Tyrode’s medium (TLH) supplemented with 0.05% (wt/vol) polyvinyl alcohol (PVA), only compact COCs with homogenous cytoplasm were chosen and rinsed three times in TLH-PVA.

### Pre-*in vitro* maturation and *in vitro* maturation

**Figure 1:**
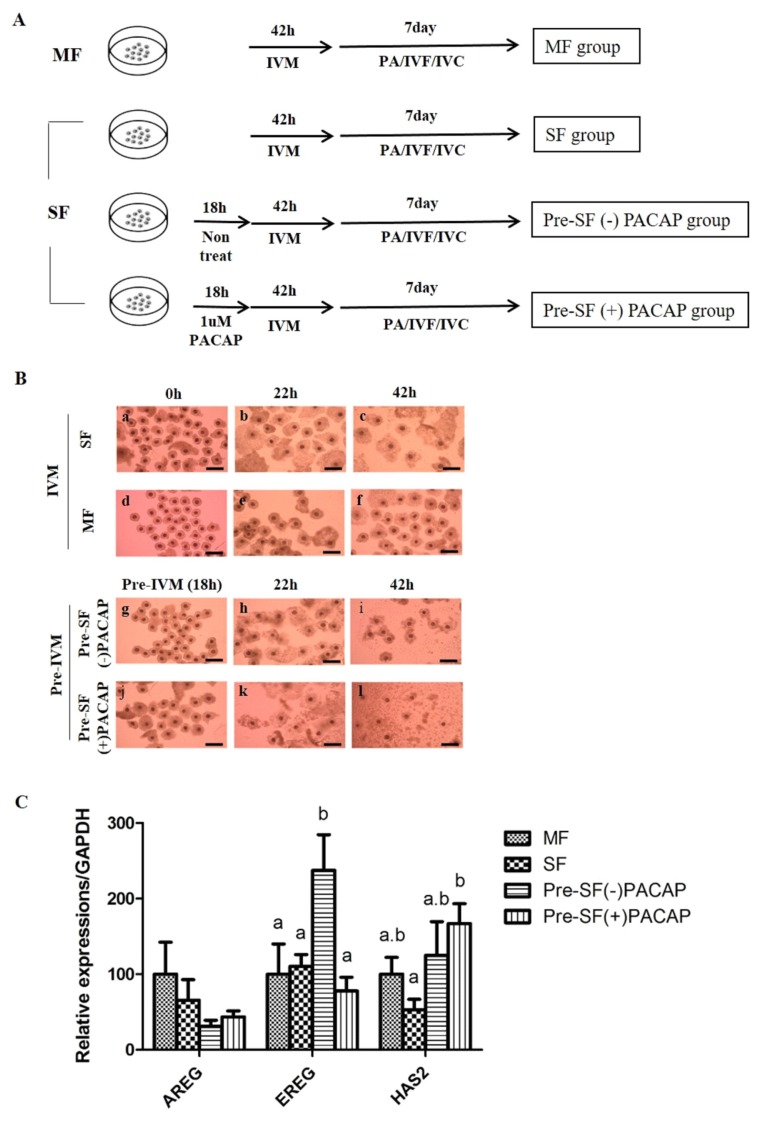
(A) Experimental design. The medium follicles (MF) group was cultured in standard *in vitro* maturation (IVM) for 42 h using cumulus-oocyte complex (COCs) derived from medium follicles. The small follicles (SF) group was cultured in the same manner as the MF group, only differing in the use of COCs derived from small follicles. Being cultured in standard IVM for 42 h following a 18 h Pre-IVM, the Pre-SF(−)PACAP and Pre-SF(+)PACAP groups were set apart according to treatment with or without 1 μM PACAP during Pre-IVM. (B) Morphological changes of COCs in accordance with time. COCs acquired from MF (a–c) and SF (d–f) were cultured for 42 h. Pre-SF(−)PACAP (g–i) and Pre-SF(+)PACAP (j–l) was cultured for 60 h, due to an addition to Pre-IVM for 18 h with or without 1 μM PACAP treatment (×100 magnification), and (C) the expression of cumulus expansion-related genes after IVM. This experiment was repeated three times. MF, medium follicles; IVM, *in vitro* maturation; COCs, cumulus-oocyte complex; SF, small follicles; PACAP, pituitary adenylate cyclase-activating polypeptide. ^a,b^ Values with differing superscripts within a column differ significantly (p<0.05).

COCs were maturated in 500 μL of pre-IVM culture medium containing TCM-199 (Invitrogen Corporation, Carlsbad, CA, USA) supplemented with 0.1% (v:v) PVA in a four-well dish (Nunc, Roskilde, Denmark). The 60 COCs isolated from SF were cultured in mediums either with or without 1 μM PACAP (Pre-SF[−]PACAP group or Pre-SF[+]PACAP group) and incubated with 5% CO_2_ in air at 39°C for 18 hours (the pre-IVM period). After the pre-IVM period, COCs were rinsed twice with the IVM composed of TCM 199 added with 0.91 mM sodium pyruvate, 0.6 mM cysteine, 75 μg/mL kanamycin, 10 ng/mL epidermal growth factor (EGF) and 1 μg/mL insulin. Then, 60 of the COCs derived from MF, SF and pre-matured groups were cultured in a medium containing with 10 IU/mL hCG and 10 IU/mL eCG at 39°C with 5% CO_2_. These groups were then maturated in a medium with hormones. After 22 hours, the COCs were rinsed twice and cultured in an IVM medium for another 20 to 22 hours. Experimental design is expressed through illustration (Figure 1A).

### Parthenogenetic activation of oocytes

Denuded mature oocytes were activated for 60 ms with two pulses of 120 V/mm of direct current during PA. The activation medium contained 0.26 mM mannitol, 0.05 mM MgCl_2_ and 0.01 mM CaCl_2_. The activated oocytes were cultured in a porcine zygote medium 3 (PZM3) mixed with 5 μg/mL cytochalasin B for 4 hours at 39°C under 90% N_2_, 5% CO_2_ and 90% O_2_. PA occurs in the methods, so be sure to confirm developmental ability of oocyte itself. The experiments were repeated four times.

### *In vitro* fertilization of oocytes

For *in vitro* fertilization (IVF), the COCs after IVM were denuded mechanically with 0.1% hyaluronidase and rinsed in TLH-PVA. After washing, approximately 15 oocytes were transferred to a Petri dish containing 40-μL microdroplets of modified Tris-buffered medium (mTBM) covered with mineral oil. Next, fresh semen transported from the Veterinary Service Laboratory (Department of Livestock Research, Yong-in city, Gyeonggi-do, Republic of Korea) was stored at 17°C before use. The semen sample was rinsed twice in Dulbecco’s phosphate buffered saline supplemented with 0.1% bovine serum albumin and centrifuged at 2,000×g for 2 minutes. Then, 5 μL of the sperm suspension was introduced to a 40 μL microdrop of mTBM as fertilization medium to yield the final sperm concentration at 1×10^5^ sperm/mL. The oocytes and spermatozoa were incubated for 20 minutes at 39°C under 5% CO_2_. After the incubation period, the loosely linked sperm cells were removed by gentle pipetting. Next, the gametes were then washed in mTBM and transferred to mTBM for 5 hours at 39°C in 5% CO_2_. IVF occurs in the methods, so be sure to confirm developmental ability through normal fertilization. The experiments were repeated four times.

### *In vitro* embryo culture

Presumptive zygotes derived the PA and IVF were washed three times with PZM3, then cultured in 30 μL microdrops (10 embryos/drop) of PZM3. The medium was covered under mineral oil and cultured in an atmosphere of 90% N_2_, 5% CO_2_, and 5% O_2_ at 39°C for 7 days. The culture media were changed at day 2 and day 4 after PA and IVF for all experiments.

### Embryo evaluation and total cell counts

On day 2, the cleavage rate of embryos was assessed using a stereomicroscope. On day 7, blastocyst formation was evaluated. In order to quantify the total cell number, blastocysts were collected and unhatched blastocysts zona pellucida (ZP) were removed with 0.5% protease. The ZP free blastocysts were rinsed with PBS, stained in 5 μg/mL Hoechst 33342 for 5 minutes and fixed in 4% paraformaldehyde. The blastocysts were placed on glass slides in 4 μL glycerol, gently compressed under a coverslip, and viewed under a fluorescence microscopy (Nikon, Tokyo, Japan) at 400× magnification. The experiment was repeated four times.

### Real-time polymerase chain reaction

Gene expression was performed on groups of cumulus cells and 200 matured oocytes. These were then sampled and stored at −80°C until analysis the gene expression levels implicated in extracellular matrix (*AREG*, *EREG*, and *HAS2*), apoptosis (*CASPASE-3*; only in oocytes, *BCL-2*, and *BAX*), cell metabolism (*HK2*; only in cumulus cells) and developmental competence (*POU5F1*; only in oocytes) were surveyed in cumulus cell and oocytes. Total RNA extraction and cDNA synthesis was carried out using SuperPrep Cell Lysis & RT kit (Toyobo Co. Ltd, Osaka, Japan) following the manufacturer’s protocol. Real-time polymerase chain reaction was performed using 1 μL cDNA template added to 10 μL 2X SYBR Premix Ex Taq (TaKaRa Bio, Inc., Otsu, Shiga, Japan) including specific primers. The program consisted of inactivation (95°C for 5 min), followed by 40 cycles of denaturation (95°C for 30 s), annealing (55°C for 30 s), and extension (72°C for 30 s). All primer sequences are presented (Table 1). The expression levels of each target gene were quantified relative to glyceraldehyde 3-phosphate dehydrogenase (*GAPDH*) and 18S ribosomal RNA (*RN18S*) expression. Threshold cycle (Ct) was used for relative quantification, at constant fluorescence intensity. The relative expression (R) was computed by the equation, R = 2–(ΔCt sample – ΔCt control). *GAPDH* or *RN18S* was used to normalize each expression value. The experiments were replicated three times.

### Statistical analysis

Statistical analyses were executed by SPSS 17.0 (SPSS, Inc., Chicago, IL, USA). A one-way analysis of variance by Duncan’s multiple range test was utilized to analyze the percentage data of cleavage and blastocyst rates, the cell numbers and relative gene expression levels. Data are given as the mean± standard error of the mean. Values of p<0.05 were regarded as be statistically significant differences.

## RESULTS

### Pre-*in vitro* maturation pituitary adenylate cyclase-activating polypeptide treatment affects cumulus expansion

Morphological changes of cumulus cell expansion are shown according to IVM step (Figure 1B). The COCs were darker in the Pre-SF(+)PACAP group than in other groups (Figure 1B). Under PACAP treatment only, some cumulus cells were attached to the bottom of the culture dish, rather than surrounding oocytes, while those around oocytes were slightly swollen. After IVM, the Pre-SF(+)PACAP group possessed fully expanded cumulus cells, indicating that pre-IVM PACAP treatment promoted cumulus expansion.

Our analysis of cumulus-expansion genes revealed that none of the groups differed significantly in AREG expression. Next, EREG expression was significantly higher in Pre-SF(−)PACAP than in MF, SF, and the Pre-SF(+)PACAP groups (p<0.05; Figure 1C). However, HAS2 transcripts were greater in Pre-SF(+)PACAP than in SF (p<0.05), even though Pre-SF(−)PACAP and Pre-SF(+)PACAP groups did not differ significantly.

### Pre-*in vitro* maturation pituitary adenylate cyclase-activating polypeptide treatment improves subsequent embryonic development

The impact of pre-IVM with PACAP on embryonic development were evaluated by PA and IVF. Pre-IVM treatment increased cleavage rate of PA embryos compared with standard IVM groups (SF and MF) (p<0.05; Table 2), with the highest rates from Pre-SF(+)PACAP (p<0.05). Post-PA blastocyst rate did not differ significantly between MF and Pre-SF(+)PACAP or between Pre-SF(−)PACAP and Pre-SF(+)PACAP. However, the MF group had significantly more total blastocyst cells than all other groups.

Following IVF, MF and Pre-SF(+)PACAP had significantly higher cleavage rates (p<0.05; Table 3). Furthermore, blastocyst rate and total blastocyst cell count were significantly higher in the MF group than in other groups, indicating greater embryonic developmental competence under MF (p<0.05). The Pre-SF(+)PACAP group experienced increased cleavage and blastocyst rates, as well as total blastocyst cell count, relative to SF and Pre-SF(−)PACAP.

### Treatment of pre-*in vitro* maturation with pituitary adenylate cyclase-activating polypeptide influences apoptosis-related gene expression in oocytes

**Figure 2:**
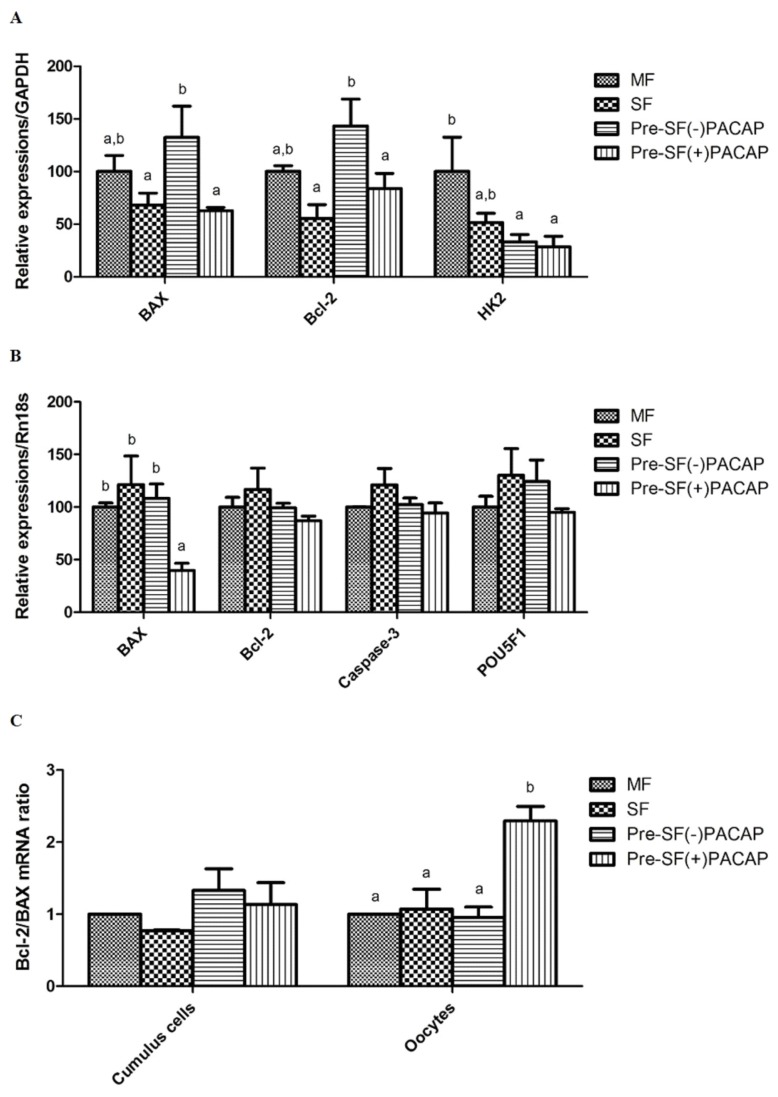
The mean±standard error of the mean expression values of mRNA in (A) cumulus cells and (B) oocytes treated with pituitary adenylate cyclase-activating polypeptide during pre-*in vitro* maturation. (C) The ratios of BCL-2 to BAX. The mount of each product was normalized to the housekeeping gene *GAPDH* and *RN18S*. BCL-2, B-cell lymphoma 2; BAX, Bcl-2-associated X; *GAPDH*, glyceraldehyde 3-phosphate dehydrogenase; RN18S, 18S ribosomal RNA. Within the same mRNA, ^a,b^ values with differing superscripts within a column differ significantly (p<0.05).

In cumulus cells, *BAX* and *BCL-2* expression did not change significantly between Pre-SF(+)PACAP and SF groups. However, Pre-SF(−)PACAP had significantly higher *BAX* and *BCL-2* expression compared with Pre-SF(+)PACAP and SF (p<0.05; Figure 2A). We also observed higher *HK2* transcript levels in MF than in either Pre-SF(+)PACAP or Pre-SF(−)PACAP (pre-IVM groups). Next, Pre-SF(+)PACAP did not differ in *BCL-2*, *CASPASE-3*, and *POU5F1* expression, but had fewer *BAX* transcripts, than the other groups (p<0.05; Figure 2B). Finally, the *BCL-2/BAX* ratio increased significantly in Pre-SF(+)PACAP oocytes, but not in cumulus cells (p<0.05; Figure 2C).

## DISCUSSION

Here, we demonstrated that pre-IVM PACAP treatment can enhance SF-derived oocyte developmental capacity. Specifically, the treatment improves embryo development and quality after both PA and IVF. Moreover, PACAP treatment upregulated cumulus-expansion genes and downregulated apoptosis genes in cumulus cells and oocytes, respectively.

Previously, we showed that pre-IVM PACAP treatment of SF-derived oocytes improves nuclear and cytoplasmic maturation [16]. Here, we observed that pre-IVM PACAP partially upregulated extracellular-matrix-formation genes. In general, oocyte maturation is triggered by luteinizing hormone or EGF-family members like EREG and AREG. When EGF-like peptides and EGF receptors bind on cumulus cells, ERK1/2 signals are activated and cumulus-expansion genes (e.g., *HAS2* and cyclooxygenase 2) are expressed [22]. Notably, *HAS2* is a known indicator of oocyte developmental capability in cumulus cells [23]. In this study, Pre-SF(+)PACAP had significantly higher *HAS2* transcript levels compared with SF, although Pre-IVM groups did not differ in the expression of this gene. We believe that PACAP treatment may explain the difference between Pre(+)PACAP and SF. In mice, PACAP treatment during IVM improves cumulus expansion because PACAP contributes to cAMP response element-binding protein (CREB) phosphorylation through the cAMP/PKA pathway [21], and CREB is important to *HAS2* expression [24,25]. Indeed, other cAMP modulators have generally increased *HAS2* expression through the same pathway [26]. Moreover, cAMP-modulator treatment can immediately upregulate extracellular-matrix-formation genes during the pre-IVM stage, although effectiveness differs across modulator type [9]. Although these results for *EREG* and *AREG* in our study are yet to be fully understood, a study in cattle also demonstrated that pre-IVM cAMP-activator treatment increased cumulus-expansion gene expression (including *HAS2*), whereas EGF-like gene expression changed during IVM in response to hormone treatment (follicle-stimulating hormone or AREG) [27]. Corroborating these earlier findings, the Pre-SF(+)PACAP group after pre-IVM in this study had swollen COCs compared with other groups. Also, the Pre-SF(+)PACAP group possessed fully expanded cumulus cells after IVM. Taking these data together, we suggest that pre-IVM PACAP treatment improves SF-derived oocyte maturation, in part through regulating genes associated with extracellular matrix formation. To maximize this beneficial pre-IVM effect, we require further research examining the link between PACAP and gonadotropins.

Pre-IVM PACAP supplementation significantly augmented developmental competence and blastocyst quality in SF-derived oocytes. Our findings address the problem of such oocytes having poor preimplantation development compared with oocytes obtained from MF and large follicles [28]. However, we should note that several *in vitro* studies failed to demonstrate that a similar pre-IVM system improved developmental potential of SF-derived oocytes compared with conventional systems [29,30]. Nevertheless, studies in cattle and sheep showed that pre-IVM treatment with a cAMP modulator increased blastocyst formation rate and quality over conventional IVM, although no differences in the production of matured oocytes following pre-IVM [31]. In this study, pre-IVM PACAP treatment caused an increase in total blastocyst cell number, indicative of blastocyst quality. The varying levels of effectiveness in pre-IVM systems may be due to interspecific variation or differences in maturation conditions. Overall, however, current findings suggest that pre-IVM PACAP treatment improves developmental competence and quality.

In the present study, we measured post-IVM expression of *BAX*, *BCL-2*, and *HK2* in cumulus cells from our experimental groups. *BAX* is a pro-apoptotic factor protein that blocks anti-apoptosis factor *BCL-2*. Elevated cumulus-cell apoptosis is associated with lower oocyte maturation rates and decreased fertilization potential post-IVF [32]. Here, we found that oocytes of the Pre-IVM(+)PACAP group had significantly lower *BAX* transcript levels. Although PACAP itself also has as an anti-apoptotic effect in neuronal and granulosa cells [33], we had previously shown that oocytes do not express the PACAP receptor. Therefore, we assumed that an increase in glutathione (GSH) largely caused *BAX* downregulation [16], given that *BAX* expression is inversely correlated with GSH [34]. Interestingly, while cumulus cells do express PACAP receptors, previous research found that PACAP treatment did not lead to significant differences in *BCL-2* to *BAX* ratios across groups [20]. We also found that *CASPASE-3* expression in oocytes did not differ across groups, despite *BAX* downregulation in Pre-SF(+)PACAP. Apoptosis mechanisms can be divided into caspase-dependent and -independent. The former is considered classical apoptosis, while the latter occurs through DNA fragmentation via apoptosis-inducing factor [35]. Clearly, further research is necessary to elucidate the exact molecular mechanism of PACAP’s pre-IVM anti-apoptotic effect.

During oocyte maturation, cumulus cells play a major role in supplying pyruvate. Unsurprisingly, therefore, glucose-metabolism genes such as *HK2* are upregulated in cumulus cells as oocytes mature. The gene is also important to ROS regulation, as well as inhibition of apoptosis and proliferation [36,37]. However, in contrast with earlier work showing that cAMP modulators increased *HK2* transcript levels [26], we found that the pre-IVM group had significantly lower *HK2* expression than the MF group, regardless of PACAP treatment. Our results could be attributable differences in follicle size, culture period, and cAMP-modulator type, all factors that influence the effectiveness of *HK2* as an apoptosis indicator. Finally, despite being a key regulator of developmental competence [38], we found no significant difference in *POU5F1* expression across groups. These experimental outcomes indicate that pre-IVM PACAP treatment may regulate apoptosis, rather than energy metabolism or development.

In conclusion, pre-IVM PACAP treatment of SF-derived COCs promoted cumulus expansion, developmental competence, embryo quality in PA- and IVF-generated embryos. Possible mechanisms include upregulation of cumulus-cell-expansion genes and downregulation of oocyte pro-apoptosis genes.

## Figures and Tables

**Table 1:** Primer sequences for analysis of mRNA gene expression

mRNA	Primer sequences	Product size (base pairs)	Accession number
*AREG*	F: 5′-TCCTCTGCTCAGCCCATTAT-3′ R: 5′-TATGTGCGGTTCGTTATCGT-3′	212	NM_214376
*EREG*	F: 5′-GCACAGCTTTAGTTCAGACAG-3′ R: 5′-TTTGCTCAGAGGTTGTTGGA-3′	206	XM_013978775
*HAS2*	F: 5′-CGGAGGAGATGTCCAGATTT-3′ R: 5′-TTGGCTGCCCATAAACTCTT-3′	211	NM_214053
*BAX*	F: 5′-TGCCTCAGGATGCATCTACC-3′ R: 5′-AAGTAGAAAAGCGCGACCAC-3′	199	XM_003127290
*BCL-2*	F: 5′-AATGACCACCTAGAGCCTTG-3′ R: 5′-GGTCATTTCCGACTGAAGAG-3′	182	NM_214285
*HK2*	F: 5′-TCAGATTGAGAGTGACTGCC-3′ R: 5′-CCCCCGGTTTTCTCGTATTT-3′	187	NM_001122987
*CASPASE-3*	F: 5′-CGTGCTTCTAAGCCATGGTG-3′ R: 5′-GTCCCACTGTCCGTCTCAAT-3′	186	NM_214131
*POU5F1*	F: 5′-GCGGACAAGTATCGAGAACC-3′ R: 5′-CCTCAAAATCCTCTCGTTGC-3′	200	NM_001113060
*RN18S*	F: 5′-CGCGGTTCTATTTTGTTGGT -3′ R: 5′-AGTCGGCATCGTTTATGGTC-3′	207	NR_046261
*GAPDH*	F: 5′-GTCGGTTGTGGATCTGACCT-3′ R: 5′-TTGACGAAGTGGTCGTTGAG-3′	207	NM_001206359

*AREG*, amphiregulin; *EREG*, epiregulin; *HAS2*, hyaluronan synthase 2; *BAX*, Bcl-2-associated X; *BCL-2*, B-cell lymphoma 2; *HK2*, hexokinase 2; *CASPASE-3*, cysteine-aspartic acid protease 3; *POU5F1*, POU Class 5 Homeobox 1; *RN18S*, 18S ribosomal RNA; *GAPDH*, glyceraldehyde 3-phosphate dehydrogenase.

**Table 2:** Effect of pre-IVM with PACAP supplementation during IVM on the embryonic development following PA

Type	No. of embryos cultured for PA(4)1)	No. (%) of embryos developed to		Total cell number in blastocyst (N)2)

		≥2-cell	Blastocyst	
MF	162	118 (74.8±0.5)^b^	73 (45.9±4.3)^c^	52.9±0.9 (20)^d^
SF	154	100 (65.8±2.9)^a^	25 (17.1±2.6)^a^	34.9±0.7 (10)^a^
Pre-SF(−)PACAP	173	143 (82.7±1.5)^c^	45 (26.4±2.2)a,b	37.6±0.6 (20)^b^
Pre-SF(+)PACAP	173	166 (95.7±0.9)^d^	64 (37.7±4.9)b,c	47.1±0.8 (20)^c^

The data are mean±standard error of the mean.

IVM, *in vitro* maturation; PACAP, pituitary adenylate cyclase-activating polypeptide; PA, parthenogenetic activation; MF, medium follicles; SF, small follicles.

1) Replication number.

2) Number of examined blastocysts.

a–d Values with differing superscripts within a column differ significantly (p<0.05).

**Table 3:** Effect of pre-IVM with PACAP supplementation during IVM on the embryonic development following IVF

Type	No. of embryos cultured for IVF(4)1)	No. (%) of embryos developed to		Total cell number in blastocyst (N)2)

		≥2-cell	Blastocyst	
MF	146	82 (56.3±1.9)^b^	33 (22.9±1.8)^c^	42.2±1.7 (15)^d^
SF	133	58 (43.6±1.1)^a^	9 (6.9±1.4)^a^	16.9±0.9 (7)^a^
Pre-SF(-)PACAP	136	63 (46.6±2)^a^	12 (9.1±2)^a^	27.3±1.9 (8)^b^
Pre-SF(+)PACAP	127	72 (56.7±2)^b^	22 (17.4±1.7)^b^	32.7±1.1 (9)^c^

The data are mean±standard error of the mean.

IVM, *in vitro* maturation; PACAP, pituitary adenylate cyclase-activating polypeptide; IVF, *in vitro* fertilization; MF, medium follicles; SF, small follicles.

1) Replication number.

2) Number of examined blastocysts.

a–d Values with differing superscripts within a column differ significantly (p<0.05).
